# Identification of molecular pathways and protein-protein interactions in adipose tissue-derived mesenchymal stromal cells (ASCs) under physiological oxygen concentration in a diabetic rat model 

**DOI:** 10.22038/IJBMS.2022.59004.13107

**Published:** 2022-02

**Authors:** Luis-Miguel Paco-Meza, MDolores Carmona, Sagrario Cañadillas, Ana Lopez-Diaz, Francisco Muñoz-López, Alvaro Jimenez-Arranz, Ipek Guler, Concha Herrera

**Affiliations:** 1 Maimonides Institute of Biomedical Research in Cordoba (IMIBIC), Spain. Avenida Menéndez Pidal s/n, CP 14004 Córdoba, Spain; 2 Cellular Therapy Unit, Reina Sofia University Hospital, Cordoba, Spain. Avenida Menéndez Pidal s/n, CP 14004 Córdoba, Spain; 3 University of Cordoba, Spain. Avenida Menéndez Pidal s/n, CP 14004 Córdoba, Spain; 4 Bio-Knowledge Lab, Glorieta de los Países Bálticos, s/n. Edificio Baobab 1, Oficina 15, Polígono Tecnocórdoba, 14014 Córdoba, Spain; # These authors contributed equally to this work

**Keywords:** Cell-based therapy, Diabetes, Enrichment analysis, Microarray, Physioxia

## Abstract

**Objective(s):**

Adipose tissue-derived mesenchymal stromal cells (ASCs) are useful in cell-based therapy. However, it is well known that diabetes mellitus (DM) alters ASCs’ functionality. The majority of *in vitro* studies related to ASCs are developed under non-physiological oxygen conditions. Therefore, they may not reflect the full effects of DM on ASCs, *in vivo*. The main aim of the current study is to identify molecular pathways and underlying biological mechanisms affected by diabetes on ASCs in physiological oxygen conditions.

**Materials and Methods:**

ASCs derived from healthy (ASCs-C) and diabetic (ASCs-D) rats were expanded under standard culture conditions (21% O_2_) or cultured in physiological oxygen conditions (3% O_2_) and characterized. Differential gene expressions (DEGs) of ASCs-D with respect to ASCs-C were identified and analyzed with bioinformatic tools. Protein-protein interaction (PPI) networks, from up- and down-regulated DEGs, were also constructed.

**Results:**

The bioinformatic analysis revealed 1354 up-regulated and 859 down-regulated DEGs in ASCs-D, with 21 and 78 terms over and under-represented, respectively. Terms linked with glycosylation and ribosomes were over-represented and terms related to the activity of RNA-polymerase II and transcription regulation were under-represented. PPI network disclosed RPL11-RPS5 and KDR-VEGFA as the main interactions from up- and down-regulated DEGs, respectively.

**Conclusion:**

These results provide valuable information about gene pathways and underlying molecular mechanisms by which diabetes disturbs ASCs biology in physiological oxygen conditions. Furthermore, they reveal, molecular targets to improve the use of ASCs in autologous transplantation.

## Introduction

Mesenchymal stromal cells (MSCs) have been proposed as a valuable tool in cell therapy due to their therapeutic effects and potential for tissue regeneration in a wide range of diseases (1-4). MSCs are undifferentiated multi-potent adult stromal cells that are self-renewable and show the capacity to differentiate into specialized lineages (5, 6). Bone marrow has been the source most evaluated and used (7) but MSCs can be isolated from other sources including adipose tissue (AT), umbilical cord blood, and periosteum, among others (5). It is well known that MSCs harvested from adipose tissue (ASCs) have some advantages such as being obtained by less invasive methods and greater proliferative capacity; moreover, they present a stable phenotype in long-term culture. Thus, their use in clinical approaches is increasing (4). But unfortunately, many studies have shown that environmental and pathologic factors impair their functionality (3, 8-10).

Diabetes Mellitus (DM) is one of the most common metabolic diseases (11). In 2017, 425 million people were affected worldwide by DM (12), and it is estimated that this figure is going to increase to 439 million people in 2030 (11, 13). Currently, pharmacological approaches to DM treatment are mainly targeted to ameliorate hyperglycemia (1, 2, 14) and improve symptoms (14) but they are ineffective in avoiding DM complications (1) and even alterations in cell functionality (15, 16). Therefore, it appears to be unavoidable that the therapeutic abilities of ASCs will be damaged by this pathological condition. For instance, it has been documented that DM impairs the gene expression profile of ASCs (17, 18), as well as, strongly disturbing their features and functionality (3, 13) due to production and accumulation of advanced glycosylated end products and the increase of oxidative stress induced by high glucose levels (8, 13), so ASCs derived from diabetic patients could be less effective in autologous cell therapies. 

Physiologically, ASCs reside in a low oxygen environment (physioxia) (9) in AT (1%-5% O_2_), far from atmospheric oxygen concentration (normoxia, 21% O_2_) commonly used in most ASCs culture protocols (5). Normoxia is a non-physiological condition and may disturb cell biology and activity (9). Therefore, the experimental studies developed under such conditions could not reflect the whole characteristics of ASCs and the full impact of other factors such as diabetes on ASCs *in vivo*. Furthermore, Nyengaard *et al*. carried out a study that unveiled the additive effects of hyperglycemia and physioxia (5% O_2_) on metabolic pathways in rat retinas (19). However, there is no knowledge about the role of culturing diabetic ASCs under physioxia. 

The gene-expression microarray is a powerful tool to measure the expression levels of a lot of genes simultaneously or to genotype multiple regions of a genome (10). Thus, DNA microarray analysis may enable the understanding and characterization of the diabetic-related ASCs dysfunction and the role of physioxia as a driver of ASCs function.

The present work aimed to reveal the molecular pathways and underlying biological mechanisms altered by diabetes in ASCs cultured under physioxia in order to enhance understanding of the effects of both factors in them *in vivo* providing new targets for improving their use in autologous cell transplantation.

## Materials and Methods

***Experimental design and DM model***

Pathogen-free 2-month-old male Wistar rats were purchased from the Experimental Animals Service (SAEX) of Cordoba University and housed at a constant temperature (20 ^º^C-22 ^º^C) with a 17/7-hr light-dark cycle. Initial rat weights were 180–200 g. On day 0, twelve rats were randomly divided into two groups (n=6 per group): healthy group (C) and DM group (D). DM was induced through intraperitoneal (IP) injection of 200 µl streptozotocin (STZ) at a dose of 60 mg/kg (20) and rats included in the healthy group were injected with 200 µl saline.

Blood samples were taken from subclavian veins after 4 hr under fasting conditions for determination of glucose levels with a Blood Glucose Test Strip (FreeStye Witney, Oxon UK) on day 0 and day 15 to verify the establishment of diabetes. Rats with a glycemia higher than 250 mg/dl were considered diabetic (21). 

Animal protocols were approved by the Institutional Animal Care and Use Committee of the Cordoba University and developed under European (86/609 / EEC, 24 November 1986) and Spanish (DOG 214/1997, of July 30) regulations for animal stabling, handling, experimentation, and other scientific purposes. All animals were treated humanely in accordance with the Guidelines established by University of Cordoba and IMIBIC and accepted by the institutional animal care and use committee of the Cordoba University.

***Isolation and expansion of ASCs***

Fifteen days after diabetes induction, AT was isolated from 6 healthy and 6 diabetic rats. For this purpose, all animals were anesthetized by IP injection of thiopental (LD_50_: 50 mg/kg) and humanely sacrificed by exsanguination through aortic puncture.

Subcutaneous AT was extracted from inguinal fat, cut into small pieces, minced, and digested in a Collagenase Type I solution (Sigma-Aldrich) at 37 ^º^C under constant agitation. The digested tissue was washed with fetal bovine serum (FBS, Gibco) to neutralize collagenase action, filtered, and centrifuged. The final cell suspension was resuspended in α-MEM (BioWhittaker® LONZA, Verviers, Belgium) supplemented with FBS 15% (Gibco), ultra-Glutamine (Bio Whittaker®), 0.1 mg/ml of streptomycin, 100 IU/ml of penicillin, and 1 ng/ml of rat-derived fibroblast growth factor-2 (rat FGF-2, Sigma-Aldrich); cell suspensions were seeded in 75 cm^2 ^culture flasks and incubated at 37 ^º^C in a humidified atmosphere containing 21% O_2 _and 5% CO_2_. Non-adherent cells were removed after three days and the culture medium was refreshed twice a week until ASCs reached 80%–90% confluence. At that moment, adherent cells were harvested by trypsinization, re-seeded in new flasks, and expanded up to passage three. At this moment flasks with ASCs derived from healthy rats (ASCs-C) and ASCs derived from diabetic rats (ASCs-D) were subjected to physiological oxygen conditions for 48 hr (3% O_2_) in a hypoxic chamber (Hypoxia Chamber Biospherix). Finally, when all cell cultures reached 80% confluence, cells were harvested by trypsinization and washed by centrifugation to conduct their characterization and genomic analysis. 

***ASC phenotype and differentiation ability***

ASCs were characterized phenotypically and functionally. The phenotype analysis was performed using the following conjugated mouse monoclonal antibodies: anti-rat CD34 (Santa Cruz, Biotechnology, Dallas, Texas, USA), CD45, and CD90 (BD Pharmigen™), and hamster monoclonal antibody anti-rat CD29 (BD Pharmigen™). Cell samples were acquired on a MACSQUANT flow cytometer (Miltenyi-Biotech, Bergisch Gladbach) and analyzed using MACS Quantify software, Version 2.5. At least 100000 events were analyzed for each marker. 

The functional characterization of ASCs was performed through differentiation potential analysis to adipocytes and osteoblasts for two weeks. For this, ASCs were seeded at a density of 100000 cells per well in a 6-well plate (BD Falcon™ Labware, Franklin Lakes, NJ, USA) and cultured with a specific culture medium (Differentiation Media Bullet Kit®-Adipogenic, LONZA; Differentiation Media BulletKits®-Osteogenic, LONZA) to stimulate adipogenic and osteogenic differentiation, respectively. Differentiated cells were fixed and stained with Oil Red O (Sigma–Aldrich, St. Louis, USA) for adipocytes and with Alizarin Red S (Sigma–Aldrich, St. Louis, USA) for osteoblasts. Differentiated cells were observed under a light microscope (Nikon Eclipse TE2000-S). Images were digitalized by NIS-Element software, version 3.2, and five digitalized images were analyzed from each stained culture.

***Microarray analysis***

The total cellular RNA was extracted from ASCs-C and ASCs-D, cultured under physiological oxygen conditions, using RNeasy Mini Kit (Qiagen) following their provided protocols. RNA quantity and quality were measured using a Nanodrop ND-1000 UV-VIS (Thermo Fisher Scientific, Inc.), and RNA integrity was assessed using the Bioanalyzer RNA 6000 Nano kit and the Agilent 2100 Bioanalyzer system, according to the manufacturer instructions (Agilent Technologies, Inc., Santa Clara, CA, USA). Total RNA from each sample was amplified and labeled following a one-color protocol (Agilent Technologies). The labeled cRNA was purified using an RNeasy Mini Kit (Qiagen Group) and cRNA concentration was measured using a NanoDropND-1000 UV-VIS (Thermo Fisher Scientific, Inc.). The microarray slide (Ref: G4858A-074036, Agilent Technologies) was hybridized using a gene expression hybridization kit (Ref: p/n 5188-5242, Agilent Technologies) following the manufacture protocol and incubated for 17 hr in constant rotation at 20 rpm and 65 ^°^C. Hybridization signals were detected using an Agilent Microarray Scanner G2565C (Agilent Technologies, Inc.). The SubioPlatform software (https://www.subioplatform.com/) (22) was used to analyze the acquired microarray images, carry out the normalization, and the subsequent data processing to identify the differentially expressed genes (DEGs). An asymptotic t-student analysis was performed with a *P*-value<0.01 and a fold-change (FC)>2.0 for up-regulated genes and <0.5 for down-regulated genes considered as significantly differentially expressed. 

***Functional enrichment analysis***

To identify the biological relevance and pathways of DEGs, a functional enrichment analysis was performed using the Database for Annotation, Visualization and Integrated Discovery (DAVID) version 6.8 (https://david.ncifcrf.gov/) (23, 24). This functional annotation tool expresses the most enriched terms. The threshold to the enrichment terms was established with an EASE score of 0.1. To identify the main functions of DEGs, Gene Ontology (GO) (http://www.geneontology.org) analysis was performed to classify genes into different hierarchical categories based on their molecular function, biological process, and cellular component (25, 26). Additionally, significant pathways of the DEGs were obtained on the basis of Kyoto Encyclopedia of Genes and Genomes (KEGG) (https://genome.jp/kegg) in order to understand the biological system functions (27, 28), and information and annotations concerning proteins were obtained from UniProt (https://www.uniprot.org) (29, 30). The Benjamini-Hochberg (BH) correction method was applied, and *P*-values were adjusted in a 0.05 cut-off.

Additionally, functional enrichment analysis was performed using clusterProfiler (31). Only enriched KEGG pathways were considered, and results were adjusted by the Benjamini-Hochberg method. *P*-value<0.05 was set as cut-off criteria.

***Protein-Protein Interaction (PPI) networks construction***

The interaction networks of the proteins identified were further explored by bioinformatics analysis. The Search Tool for the Retrieval of Interacting Genes/Proteins (STRING; v11.0) (https://string-db.org) (32, 33) was used to construct the PPI network. An extended network was constructed by setting the required combined score (CS) to >0.7. The number and values >0.7 of evidence channels (EC) related to its respective CE were considered to support CE. There are seven ECs: experiments channel, database channel, text mining channel, coexpression channel, neighborhood channel, fusion channel, and the co-occurrence channel. In the network, proteins were represented by nodes, and interactions between proteins were represented by edges.

***Statistical analysis (except microarray and enrichment analysis)***

All data were expressed as mean±standard error of the mean (SEM). Because of the small sample size, non-parametric statistical analysis was used. Comparisons between two groups were made with the Mann-Whitney U test. Statistical significance was accepted when the *P*-value was ≤ 0.05. PASW Statistic 18 (IBM SPSS) software package was used to perform statistical analysis of the *in vitro* assays. 

## Results

***Induction of diabetes***

**Figure 1:**
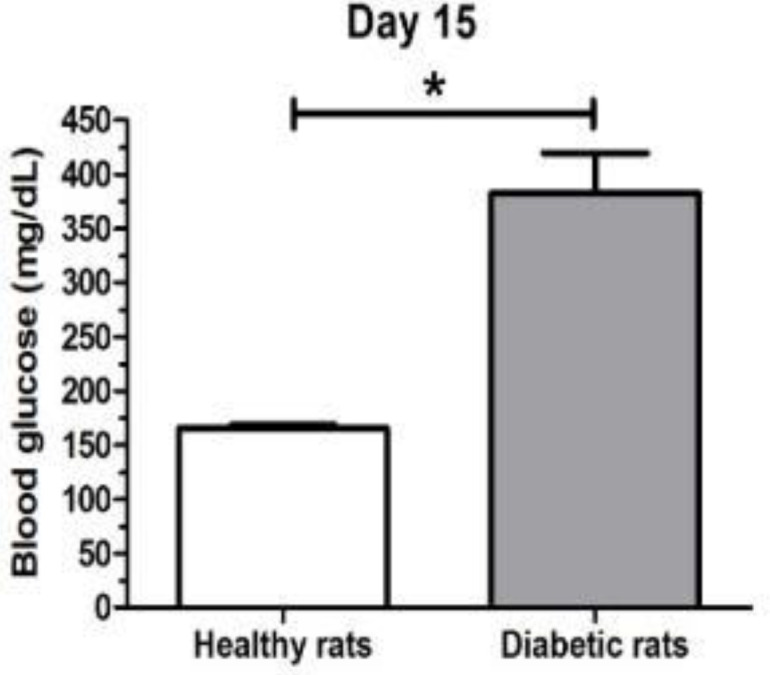
Blood glucose levels

On day 0, blood glucose levels corresponded with values of healthy animals (172.8±12.48 mg/dl). Fifteen days after STZ-injection, blood glucose levels of diabetic rats (382.6±37.15 mg/dl) were significantly higher than healthy rats (166±2.67 mg/dl) (*P=0.05*) (Figure 1). 

***Phenotypic and functional characterization of ASCs***

**Figure 2:**
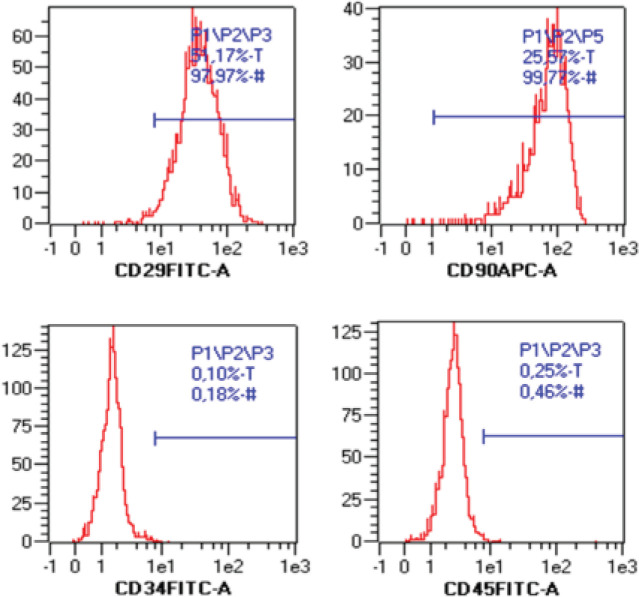
Flow cytometry analysis of adipose tissue-derived mesenchymal stromal cells (ASC): Histogram graphs derived from immunophenotypic analysis of ASCs

ASCs-D and ASCs-C cultured under physiological oxygen conditions were phenotypically homogeneous to both positive (CD29 and CD90) and negative markers (CD34 and CD45) (Table 1). Figure 2 shows a representative histogram of the flow cytometry analysis. 

Functional characterization of ASCs showed that both ASCs-D and ASCs-C were able to differentiate into adipocytes and osteoblasts after two weeks of culture in adipogenic or osteogenic inductive media (data not shown).

***Functional and pathway enrichment analysis***

The microarray analysis unveils that under physiological oxygen conditions there are as many as 2213 DEGs (1354 up-regulated and 859 down-regulated) in ASC-D in comparison to ASC-C**.** A total of 21 and 78 terms were significantly enriched for up-regulated and down-regulated DEGs, respectively, based on the setting threshold of Benjamini-Hochberg <0.05. The 12 most significant over- and under-represented terms are shown in Tables 2 and 3, respectively.

**Figure 3:**
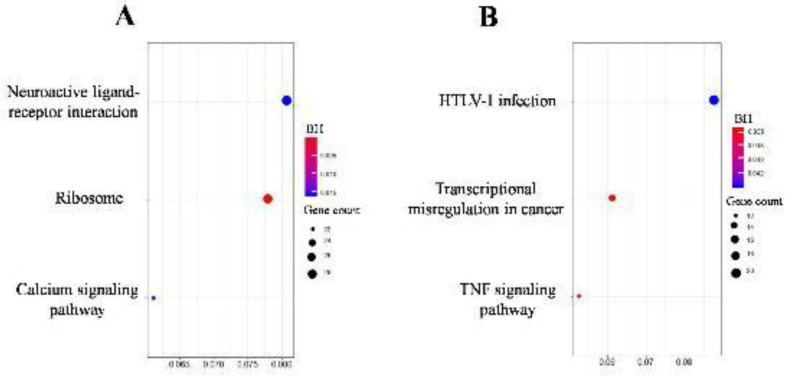
ClusterProfile derived from the Kyoto Encyclopedia of Genes and Genomes (KEGG) over-represented (A) and under-represented (B) pathways carried out with diabetic-induced adipose-tissue derived mesenchymal stromal cells regarding healthy adipose-tissue derived mesenchymal stromal cells (ASCs-D vs ASCs-C). The dot size is according to the number of genes linked with its respective pathway. The Benjamini-Hochberg (BH) adjusted P-values were arranged from less (blue) to more (red) significant. HTLV-1 (Human T- cell leukemia virus type 1), TNF (Tumor necrosis factor)

Results derived from the functional enrichment analysis performed with clusterProfiler tool recognized significant pathways based on the KEGG database according to DEGs interactions and functions. A total of 3 over-represented and 3 under-represented significant pathways were identified *(*Figure 3*)*.

***PPI networks***

**Figure 4:**
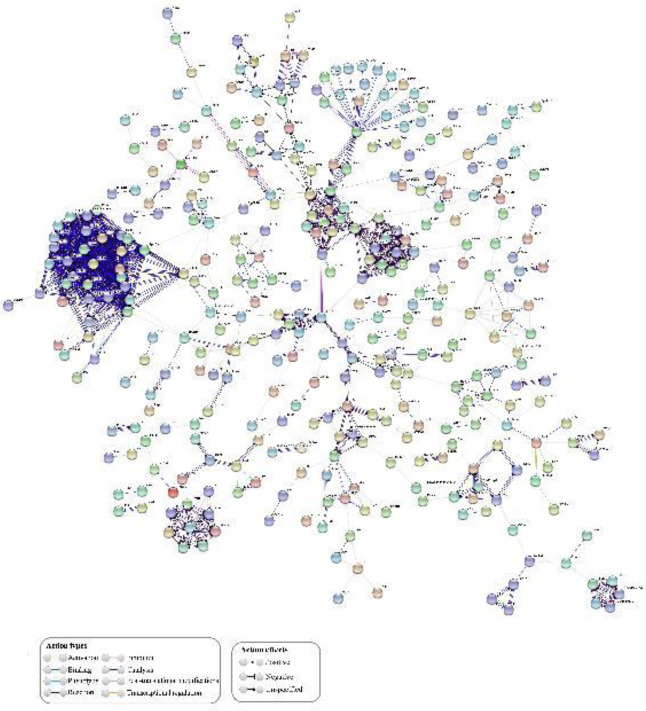
Protein-Protein Interaction (PPI) analysis of up-regulated differential gene expression (DEGs) performed in diabetic-induced adipose-tissue derived mesenchymal stromal cells regarding healthy adipose-tissue derived mesenchymal stromal cells (ASCs-D vs ASCs-C). The color of the line represents action types and the line configuration indicates action effects

**Figure 5:**
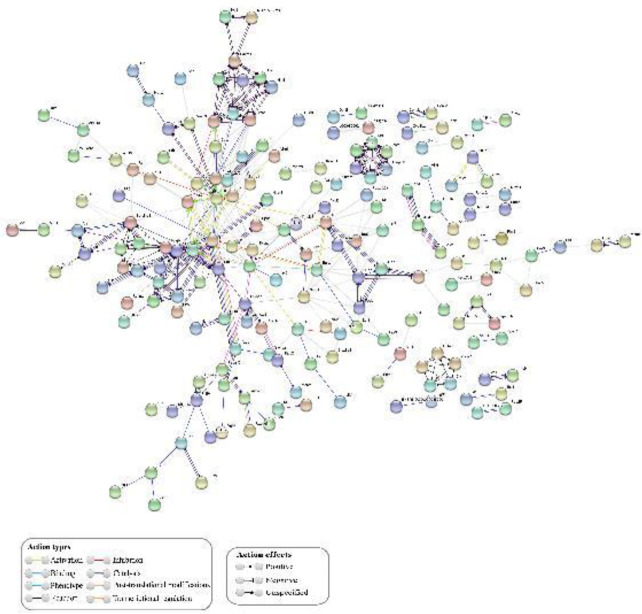
Protein-Protein Interaction (PPI) analysis of down-regulated differential gene expression (DEGs) performed in diabetic-induced adipose-tissue derived mesenchymal stromal cells regarding healthy adipose-tissue derived mesenchymal stromal cells (ASCs-D vs ASCs-C). The color of the line represents action types and the line configuration indicates action effects

The network derived from the up-regulated DEGs comprised 355 nodes, each of which represents a protein, and 891 interactions (Figure 4*).* RPL11-RPS5 interaction obtained the highest combined score reaching 0.99 and two evidence channels (EC) that attained values of 0.92 (coexpression channel) and 0.97 (experiments channel). The network derived from the down-regulated DEGs showed 185 nodes and 314 interactions (Figure 5) where KDR-VEGFA interaction was on the top with a CS=0.99 and two EC with values of 0.95 (text mining channel) and 0.9 (database channel). 

## Discussion

*In vitro* expanded MSCs have been widely used in a broad range of clinical trials in regenerative medicine (4). It is well documented that diabetes induces failure in MSCs functionality and alters their gene expression profile (3, 17). However, little is still known about the effects of DM in ASCs biology under physiological oxygen conditions. Therefore, in this study, we evaluated the gene expression profile of diabetic ASCs in relation to healthy ones under physiological oxygen conditions through DNA microarray. In this sense, the results obtained in the current study provide new insights into the understanding of ASCs’ biology at the molecular level in order to optimize their clinical outcomes. 

Nonetheless, as with the majority of studies, the experimental design of the current work is subjected to limitations. The major limitation of this research is the lack of molecular validation through a specific technique. However, despite this, our study offers new useful information for the scientific community and could address future investigations. 

Our results confirm the establishment of diabetes in STZ-induced rats (21) and the cellular identity of ASCs analyzed in the current study (i.e., immunophenotype and differentiation ability) (5) as expected.

The functional enrichment analysis showed 99 significant terms identified through GO, KEGG, and UniProt databases: 21 terms over-represented and 78 terms under-represented in ASC-D. One term of particular interest among the 21 over-represented terms is the “Glycosylation site N-linked”. Recent studies have shown that inadequate glycemic control is directly associated with N-glycan profile modifications in sera of type 1 diabetic patients (34). Furthermore, the N-glycosylation profile of MSCs surface is modified throughout their development stages (35), and changes in N-glycan processing in MSCs might affect their functional fate and differentiation capacity (36). According to these observations, based on the fact that high glucose levels produce alterations in colony formation and differentiation capacities of MSCs (8), we might suggest that diabetes could affect the behavior of ASCs through modification of the surface N-glycosylation profile and processing of N-glycans.

Interestingly, ten of the top 12 over-represented terms in ASC-D contain in their gene list, provided by enrichment analysis, two N-glycosylated genes, *Fgfr4* (37) and *Il-1r2* (38). *Fgfr4* codifies the fibroblast growth factor receptor 4 whose up-regulation has been demonstrated to have a role in glucose metabolism in cancer cells (39) suggesting that *Fgfr4* could also play a role in ASCs-D glucose metabolism. On the other hand, *Il-1r2* gene encodes the interleukin 1 receptor 2. IL-1 is the ligand to both IL-1R2 and IL-1R1 that acts as negative and positive effectors over the IL-1 signaling pathway, respectively (40). IL-1 promotes the anti-inflammatory effects of MSCs by binding IL-1R1 (41, 42). Moreover, it has been also described that ASCs derived from type 2 diabetic patients have a reduction in their immunomodulatory effects through less effectiveness in suppressing B-cell and T-cell proliferation, among other mechanisms (18). According to these published data, we suggest that dysregulation of IL-1R2 is involved in the reduction of anti-inflammatory properties of diabetic ASCs under physiological oxygen conditions.

In relation to the over-represented terms “Ribosome” and “Ribosomal protein”, and the over-represented “Riboseme Pathway”, identified in the functional enrichment analysis preformed in ASC-D, and as has been reported by published studies, high glucose levels increase the capacity of mRNA translation and protein synthesis by inducing ribosome biogenesis in kidney glomerular epithelial cells (43). This increment of mRNA activity induces a misbalance between synthesis and degradation of proteins thus producing accumulation of proteins in the extracellular matrix and leading to structural and functional alterations in the kidney culminating in diabetic renal complications (44). Diabetes might disrupt stem cells’ homeostasis and negatively alter their niche (16). In this scenario, we speculate that diabetes could induce microenvironmental alterations in the ASCs niche and disturbance in ribosomes and protein synthesis.

Regarding the 78 significantly under-represented terms, “Positive regulation of transcription from RNA polymerase II promoter”, “Transcription from RNA polymerase II promoter” and “Positive regulation of gene expression” resulted among the most significant terms after functional enrichment analysis of DEGs. All of them share the common characteristic of being actively involved in the positive regulation of the transcription process. These observations are in line with the results obtained from a study developed by Meugnier *et al*. where it was demonstrated that acute hyperglycemia modifies the adipose tissue and skeletal muscle gene expression in healthy subjects and that down-regulation of genes related to almost all biological pathways represented more than 80% of these changes (45).

Among the significantly under-represented terms, the *Nr4a1* gene (*orphan nuclear receptor 4A1*), was found in seven terms: “Positive regulation of transcription from RNA polymerase II promoter”, “Nucleus”, “Transcription from RNA polymerase II promoter”, “Cytoplasm”, **“**Sequence-specific DNA binding”, “Transcription regulation” and “Transcription”. NR4A1 is, among others, a pro-angiogenic protein that is known for being a modulator of the vascular endothelial growth factor A (VEGF-A) (46). Moreover, Yoo *et al*. after exposing tumor cells under low oxygen conditions (0.1% O_2_) unveiled the important role of NR4A1 in stabilizing the hypoxia-inducible factor (HIF) (47), which in turn, plays a pivotal role as a cell regulator in response to a low oxygen environment. In fact, it is thought that the beneficial effects of low oxygen tension on ASCs activity, such as expression of antiapoptotic proteins and enhancement of angiogenic paracrine secretion, are mainly attributed to HIF action (5, 9). Several studies carried out under atmospheric environment have revealed that diabetic MSCs possess impaired angiogenic properties since their expression of vessel formation factors, for instance, Vegfa and angioprotein-2 are lower than in cells isolated from healthy donors (6) furthermore, *in vitro* experiments showed their weaknesses in forming vessels (6, 48). Therefore, we speculate that dysregulation of *Nr4a1* could be another mechanism responsible for the disturbances of ASCs properties mediated by diabetes, and because of its action over HIF the alterations in *Nr4a1 *expression may be more notorious under physioxia and could represent a useful target to improve angiogenesis capacity of ASCs *in vivo*.

From the functional enrichment analysis performed with the clusterProfiler tool, the “HTLV-1 Infection” pathway resulted in one of the significantly under-represented pathways. It is documented that HTLV-1 (Human T- cell leukemia virus type 1) is an oncovirus responsible for an aggressive CD4 T cell malignancy in which the oncoprotein Tax besides inducing its pathogenicity, also promotes the expression of genes related to migration and angiogenesis in MSCs (49). Moreover, a study identified a Tax-responsive element (TRE), located within the nitric oxide synthase (NOS) promoter, which induces the response to hypoxia stimuli in endothelial cells by augmenting NOS production even in uninfected cells (50). These observations might unveil another possible mechanism by which diabetes alters the normal ASCs functionality and angiogenic properties.

Aiming to demonstrate the interaction of the proteins encoded by the DEGs and to analyze their biologic function, PPI networks were constructed from up-regulated and down-regulated DEGs using STRING analysis. The PPI network derived from the up-regulated DEGs showed the RPL11-RPS5 protein interaction as the most representative. RPL11 is a ribosomal protein that induces apoptosis via p53, by directly associating with its physiological repressor mouse double minute 2 (MDM2). The ribosomal protein –MDM2-p53 axis has been described as a protector against genomic instability, cancer, and alterations of ribosome-impaired biogenesis triggered by cellular stress (51, 52). It has not been studied in the case of DM, but we can hypothesize that DM provokes disturbances in ribosome biogenesis, as a consequence of hyperglycemia, inducing ASCs stress which in turn produces an accumulation of free fractions of RPL11, and their increased susceptibility to undergo apoptosis.

The PPI network derived from the down-regulated DEGs showed the KDR-VEGFA protein interaction as the most representative. It is well known that this axis constitutes the main ligand-receptor interaction that regulates cell proliferation, migration process, and angiogenesis (53) in some cellular types. This result at the protein interaction level is in agreement with the study carried out by Kin *et al*. who observed a down-regulation of Vegfa in diabetic MSCs isolated from rat bone marrow (6). 

**Table 1:** Adipose tissue-derived mesenchymal stromal cell (ASC) immunophenotypic characterization by flow cytometry. Data represent mean±SEM

***Cell populations***	***% CD29***	***% CD90***	***% CD34***	***% CD45***
ASCs-C*	98.35±1.23	99.78±0.04	0.15±0.03	0.52±0.17
ASCs-D*	98.88±0.3	98.14±0.63	0.12±0.04	0.45±0.16

*ASCs derived from healthy (C) and diabetic (D) Wistar rats (p=ns)

Blood glucose serum concentration after fifteen days of saline or streptozotocin injection in healthy and diabetic rat groups, respectively. *P=0.05. Data represent mean ± SEM

**Table 2:** Over-represented terms derived from the functional enrichment analysis

**Category**	**Term**	**Gene count**	**P-value**	**Fold Enrichment**	**BH**
**UP_SEQ_FEATURE**	Topologicaldomain: Cytoplasmic	121	2.96E-11	1.76	3.28E-08
**UP_SEQ_FEATURE**	Topologicaldomain: Extracellular	98	7.10E-10	1.83	3.94E-07
**KEGG_PATHWAY**	rno03010: Ribosome	28	2.50E-08	3.46	6.50E-06
**UP_KEYWORDS**	Disulfide bond	148	2.63E-08	1.57	8.29E-06
**UP_SEQ_FEATURE**	Transmembrane region	143	4.53E-08	1.48	1.67E-05
**UP_KEYWORDS**	Glycoprotein	140	2.37E-07	1.53	3.73E-05
**UP_KEYWORDS**	Signal	204	4.12E-07	1.39	4.33E-05
**UP_SEQ_FEATURE**	Glycosylation site: N-linked (GlcNAc,)	125	6.25E-07	1.48	0.00017335
**UP_SEQ_FEATURE**	Disulfide bond	95	1.08E-06	1.60	0.00023935
**UP_KEYWORDS**	Ribosomal protein	28	1.66E-05	2.54	0.00131022
**UP_SEQ_FEATURE**	Signal peptide	103	2.75E-05	1.46	0.0050655
**UP_KEYWORDS**	Transmembrane helix	319	6.33E-05	1.20	0.0039812

Kyoto Encyclopedia of Genes and Genomes (KEGG), Uniprot (UP), Benjamini-Hochberg (BH).

**Table 3:** Under-represented terms derived from the functional enrichment analysis

*Category*	*Term*	*Gene* *count*	*P-value*	*Fold enrichment*	*BH*
*GOTERM_BP_DIRECT*	GO:0045944~Positive regulation of transcription from RNA polymerase II promoter	72	1.39E-13	2.61	3.86E-10
*GOTERM_BP_DIRECT*	GO:0000122~Negative regulation of transcription from RNA polymerase II promoter	51	2.54E-09	2.54	3.51E-06
*GOTERM_CC_DIRECT*	GO:0005634~Nucleus	189	4.61E-09	1.45	1.63E-06
*UP_KEYWORDS*	Nucleus	110	8.54E-09	1.73	2.31E-06
*GOTERM_BP_DIRECT*	GO:0006366~Transcription from RNA polymerase II promoter	34	1.06E-08	3.17	9.73E-06
*GOTERM_CC_DIRECT*	GO:0005737~Cytoplasm	198	1.83E-08	1.41	3.24E-06
*GOTERM_MF_DIRECT*	GO:0043565~Sequence-specific DNA binding	41	7.21E-08	2.59	4.69E-05
*GOTERM_BP_DIRECT*	GO:0008284~Positive regulation of cell proliferation	37	9.80E-08	2.73	6.78E-05
*GOTERM_BP_DIRECT*	GO:0010628~Positive regulation of gene expression	30	2.92E-07	3.00	0.000161789
*UP_KEYWORDS*	Transcription regulation	47	1.65E-06	2.15	0.000223086
*GOTERM_MF_DIRECT*	GO:0003700~Transcription factor activity, sequence-specific DNA binding	45	2.50E-06	2.15	0.000811934
*UP_KEYWORDS*	Transcription	48	3,40E-06	2.07	0.00030701

Annotation of Gene Ontology (GO), Kyoto Encyclopedia of Genes and Genomes (KEGG), Uniprot (UP), Benjamini-Hochberg (BH). GO Term: CC: Cellular Component, BP: Biological Process, MF: Molecular Function

## Conclusion

In summary, the present work provides remarkable previously unpublished information regarding the diabetic-derived ASC gene expression modifications induced by hyperglycemia in physiological oxygen conditions and shows potential targets to improve the autologous transplantation use of ASC in cell-based therapy. Some important biological processes and pathways involved in glycosylation, ribosomes, positive regulation of the transcription process HTLV-1 Infection, as well as protein interactions such as RPL11-RPS5 and KDR-VEGFA could be closely associated with diabetes regarding the functionality of ASC in physiological oxygen conditions. Nonetheless, specific additional molecular studies are required to substantiate our observations and to establish whether these results are replicated in humans.

## Authors’ Contributions

LMPM, MDC, SC, and CH provided study conception or design; LMPM, MDC, SC, and ALD analyzed the data and prepared the draft manuscript; AJA, IG, and CH critically revised the paper; CH supervised the research; LMPM, MDC, SC, ALD, FMML, AJA, IG, and CH approved the final version to be published.

## Conflicts of Interest

The authors declare there are no conflicts of interest.
